# Measuring the Elusive Half-Life of Samarium-146

**DOI:** 10.1021/acscentsci.4c02221

**Published:** 2025-01-13

**Authors:** Mara Johnson-Groh

In May 2016, close to the northern border of Switzerland, radiochemist Zeynep Talip suited up for the hot laboratory (Hotlab) at the Paul Scherrer Institute (PSI), where experiments with radioactive samples can be safely executed. After donning a full-body white protective suit and dosimeter, Talip began work that had been years in the making.

In the previous months, she had honed a chemical separation technique for extracting a sizable sample of radioactive samarium-146 from a chunk of nuclear waste. She had practiced with a nonradioactive substitute, making sure every step was optimized and could be executed efficiently. Now that she was finally ready for the real sample, she could be confident that radiation risks were minimized.

**Figure:**
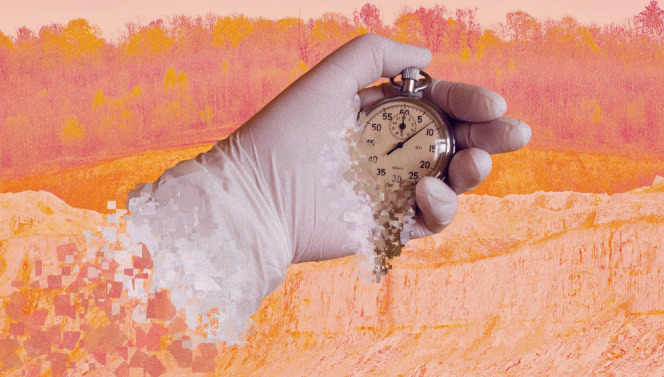
Credit: Madeline Monroe/C&EN/Shutterstock.

The work Talip and her collaborators were undertaking at the Hotlab was the first step in measuring the half-life of samarium-146. This long-lived isotope and its stable decay relic, neodymium-142, are essential for dating ancient rocks to determine the age of Earth’s crust, the moon, Mars, and other objects. Unlike some long-lived isotopes, whose concentrations in rocks can be altered by physical or chemical processes after the rock forms, samarium-146 is not significantly affected by geological processes; it changes notably only as it slowly decays to neodymium-142.

This means the samarium-146 isotopes can be used as more reliable age markers for ancient rocks than other isotopes, which may have been altered by geologic activity. When scientists go to date an ancient rock, they use the estimates of samarium-146’s half-life to calculate when the rock solidified out of magma.

“When we say we think we know the age of Mars, or the age of the moon, it’s largely based [on these isotopes],” says geochronologist Lars Borg of the Lawrence Livermore National Laboratory (LLNL).

But for over half a century, experimental half-life measurements varied widely, with results spanning more than 30 million years. This meant the ages of certain rocks could be off by tens of millions of years, making it harder for scientists to understand the events that shaped Earth and other celestial bodies shortly after their formation.

To resolve these discrepancies, Talip and a group at the PSI turned to a new technique they developed to extract large samples of samarium-146 and other scientifically important isotopes from nuclear waste. Their newly published measurements, plus more on the way from other scientists developing unique techniques for measuring half-lives, are finally closing in on an accurate number. And in addition to helping to clarify the history of the cosmos, these efforts are leading to advances in medical research, nuclear technology, and the search for some of the universe’s most elusive matter.

## Era of errors

In theory, determining a half-life is simple. It comes down to a basic calculation of two key measurements: the rate at which an isotope decays and how many atoms are present at the time of testing. Once these measurements are made, researchers simply divide the number of atoms by the decay rate and take a logarithm to get the half-life.

But making consistent half-life measurements has proved difficult for many long-lived isotopes, including samarium-146. In the 2000s, an international collaboration set out to rigorously measure the half-life of samarium-146 and reconcile the conflicting measurements from decades earlier, when laboratory equipment was less advanced. In 2012, they reported in *Science* that the half-life was 68 million years—about 30% less than previous values. But that value, which clashed with pieces of geological evidence, perplexed some geologists. It would take more than a decade to resolve the confusion.

The first inklings that something was amiss came in 2023. It was a typical day at the office for Michael Paul, coauthor on the 2012 paper and physicist at the Hebrew University of Jerusalem. But when he opened his inbox, he found a surprising email from another scientist. It claimed that the 2012 research contained a small yet significant error. He was shocked.

The issue came down to the amount of samarium-146 in the experiment. Hatched in supernovas and violent cosmic explosions involving neutron stars, the isotope no longer occurs naturally on Earth, so researchers studying it must create their own through tedious processes that typically yield very little.

The sample of samarium-146 Paul and his colleagues created was too small to allow a measure of the number of atoms, so it was diluted with a small amount of the more readily available samarium-147. But something had gone wrong in measuring the dilution with mass spectrometry. The error resulted in an incorrect value for the number of samarium-146 atoms, which threw off the calculated half-life. Paul and his colleagues retracted the paper in 2023.

Even before the retraction, there had long been calls for better measurements of samarium-146 and other geologically important isotopes due to sizable spans in reported half-lives. In 2009, a joint task group was formed to conduct an extensive review assessing the legitimacy of past experiments and to recommend standard half-lives for important isotopes, including uranium, rubidium, and of course samarium. Upon careful evaluation of samarium-146 experiments, it was clear that errors had long predated Paul’s work. Some experiments miscounted decays, while others incorrectly counted the number of atoms present.

“Invariably, all subjective evaluations of the uncertainties by the authors were off—too low,” says geochemist and task group leader Igor Villa of the University of Bern and the University of Milano-Bicocca. “There were hidden systematic flaws in the experimental designs, which affected the results.”

As a result, the group couldn’t recommend a single standard value for samarium-146’s half-life. Instead, in 2020, they recommended two, one of which was the now retracted value calculated by Paul’s team. When dating a rock, geologists would do their calculations twice—once with each recommended half-life—and report two potential dates for the rock’s age. Clearly, a better measurement was needed.

## Old problem, new approaches

Typically, creating samarium-146 involves irradiating a sample of a heavy metal, such as tantalum, with a powerful proton accelerator. When the accelerator’s high-energy protons strike the atoms in a sample, it causes nuclear reactions that eject nucleons from the sample to create new isotopes. Scientists can produce samarium-146 by tuning the acceleration of the beam of protons to specific energies, which creates isotopes in a target range of masses. The new samarium atoms, smattered throughout the metal, must then be chemically extracted from the tantalum and other byproducts.

**Figure:**
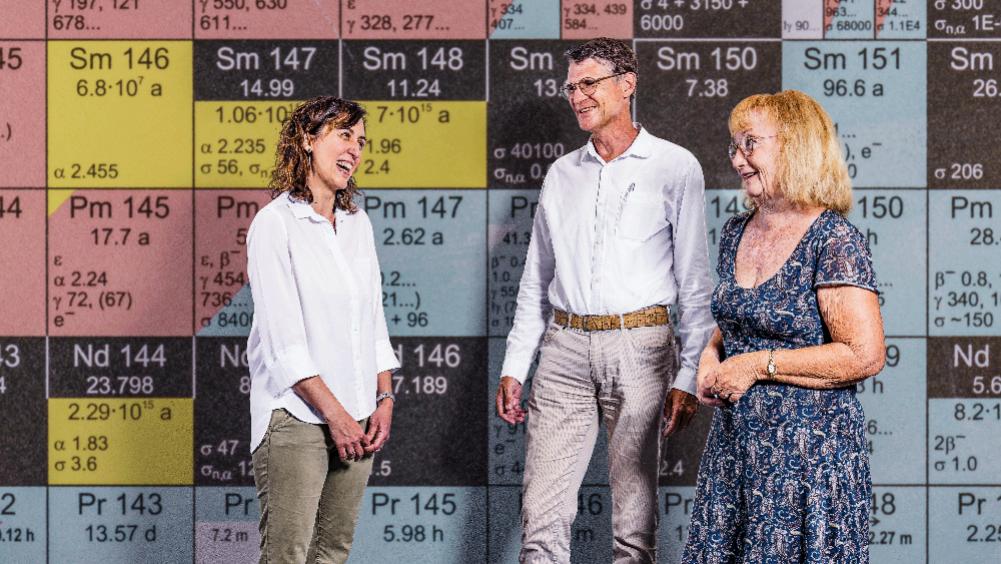
(From left) Zeynep Talip, Rugard Dressler, and Dorothea Schumann of the PSI team worked for years to improve the half-life measurement of samarium-146. Credit: Paul Scherrer Institute.

In the spring of 2016, Talip was trying to circumvent this costly process. Instead of irradiating a new sample, Talip started with a piece of leftover tantalum that had been irradiated in an experiment 17 years prior. In that experiment, the irradiation had produced practically the whole periodic table up to tungsten—including samarium-146. So instead of creating the samarium from scratch, all Talip had to do was extract it from the waste left behind in those experiments.

This approach was spearheaded by Dorothea Schumann, retired chemist and former head of the Isotope and Target Chemistry research group in the Laboratory for Radiochemistry at PSI. In the 2000s, she realized it was possible to distill the unwanted nuclear waste left over from previous radiation experiments into pure samples of rare isotopes useful in a range of experiments.

She launched the Exotic Radionuclides from Accelerator Waste for Science and Technology (ERAWAST) initiative, and over the next 2 decades, her group produced samples of gadolinium, terbium, dysprosium, and more, to measure their half-lives. They had also extracted isotopes for other uses, such as developing nuclear medicine imaging tests, environmental dating, and experiments that help scientists understand the cosmic origins of heavy elements.

“We don’t need any extra time for making these isotopes; they are already produced,” Schumann says. “The challenge is to develop the chemistry to get it out in the quantity and also quality.”

This extraction, however, is still a challenging endeavor. The technique developed by Talip and the PSI group involved first dissolving the tantalum sample in a solution of nitric acid and hydrofluoric acid—a process that alone took her 2 days in the Hotlab. Next came a complex multistep chemical separation procedure, also completed in the Hotlab, to slowly and carefully precipitate out the samarium, free from impurities.

“The devil is in the details,” Talip says. “You have to really take care with all the parameters. You should be a good analytical chemist if you want to have reproducible results.”

Including the time outside the Hotlab developing the separation technique, it took almost 3 years of work before the extracted sample of samarium-146 from Talip’s team was ready to be tested. To ensure that it was pure, they used an inductively coupled plasma mass spectrometer (ICP-MS), which atomizes a sample to determine its contents. A γ spectrometer ensured that the sample had enough radioactivity to measure the half-life.

“The [ICP-MS] results showed the samples were really pure,” says Talip, who now leads the Isotope and Target Chemistry research group at PSI.

Ultimately, Talip and her collaborators produced 1.5 μg of samarium-146—what they’ll tell you is a big amount. Schumann compared this amount to what Marie Curie had accomplished over 120 years ago when she took several metric tons of pitchblende, a uranium-containing mineral, to produce 0.1 g of radium. The ratio of raw material to pure sample has remained about the same, but since measurement techniques have improved over the century since Curie’s work, much less material is needed today.

**Figure:**
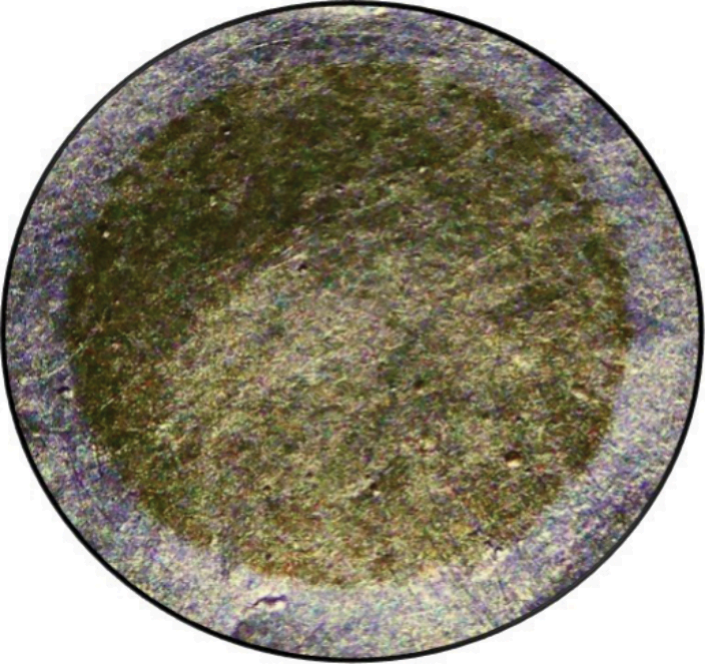
Sample of samarium-146 prepared by the PSI group. Credit: Rugard Dressler.

With a confirmed pure sample in hand, Talip’s group turned to measuring the decay rate and amount of sample. Measuring the decay rate is relatively straightforward. It involves creating a thin layer of samarium on a chemically inert film and using an α radiation detector to measure the number of decays in a set time frame. But much care still needs to be taken to ensure that no decays are missed and no external effects, like cosmic rays, present false decay detections.

To help avoid a systematic error that had befallen previous experiments in measuring the number of atoms, the group decided to send a sample of the samarium to another laboratory to independently verify the number. When the results came in, they were in perfect agreement with the PSI group’s measurements.

After extensive checking, the PSI group published their results in August 2024. They reported a half-life for samarium-146 of 92 million years, with an uncertainty spanning less than 3 million years. Their work has been positively received in the scientific community. But given the field’s checkered history, some are hesitant to accept the results without additional verification.

“My gut feeling is that the [PSI] experiment is excellent, but I also thought that [2012] experiment was well documented, and it wasn’t,” Villa says. “My personal feeling favors the 100 million years ballpark, but it’s a personal opinion. It’s not, repeat *not*, the [joint task group’s] recommendation.”

The task group is waiting on results from other ongoing experiments before they recommend a new standard samarium-146 half-life. The PSI group is also eagerly awaiting those results, even as they work on replicating their own experiment to further reduce their uncertainties.

“We are absolutely convinced that our measurement was performed in the best way we can do_⋯_ But maybe we overlooked something—this can happen,” says Rugard Dressler, coauthor on the 2024 paper and chemist at PSI’s Laboratory for Radiochemistry. “This is the reason why we are now looking very enthusiastically forward to other experiments which also tackle the determination of this half-life.”

## Double- and triple-checking

One of the experiments in the works to confirm samarium-146’s half-life is Paul’s. Today, he’s working with the same collaborators from Japan and the US as before, using some of the samarium left over from their 2012 work. Taking time to ensure that they avoid the errors of their past, they expect results no earlier than the end of this year.

Another group at the LLNL is nearing publication of their own results determined with a new technique. Instead of using a radiation detector to count the number of decays coming from a sample, they used a cryogenic detector. With this method, a sample of samarium-146 is cooled to just a few millikelvin to measure the heat created as the atoms decay. Physicist Alexander Kavner, a postdoctoral fellow at the University of Zurich who worked on the LLNL project for his doctorate, says this method is more sensitive and less susceptible to overestimating the number of decays.

“It’s next to impossible for our method to overestimate the half-life,” Kavner says.

As with the PSI group’s work with nuclear waste, applications for LLNL’s supercooled technique are also extending beyond geochronology. Using this cryogenic technology, Kavner is now developing better detectors for measuring a theoretical form of dark matter that scientists refer to as weakly interacting massive particles, or WIMPs. No one yet knows what dark matter is made of, but it is known that this evasive stuff hardly interacts with normal matter and makes up for some 85% of the total matter in the universe. Kavner’s detectors could measure WIMPs lighter than those that other experiments have been able to thus far.

Additionally, the new cryogenic approach spearheaded by LLNL could be used to better measure radiation backgrounds in fields like environmental monitoring and medical physics, where knowing the levels of radiation dosage is critical for patient health.

As for their results with samarium-146, the LLNL group has found an age of 86 million years, as Kavner reported in his graduate dissertation. He says this result is within 2 standard deviations—a confidence level of 95%—of the PSI findings. Ultimately, if the half-life can be narrowed down, it will give geochronologists a better chance of understanding our solar system’s history. But despite the recent advances, there’s still some way to go before the story of Earth’s past can be written in stone.

*Mara Johnson-Groh is a freelance contributor to*Chemical & Engineering News*, the independent news outlet of the American Chemical Society.*

